# Editorial: Cardiomyocyte Maturation: Novel Insights for Regenerative Medicine

**DOI:** 10.3389/fcell.2021.730622

**Published:** 2021-08-02

**Authors:** Min Zhang, Li Qian, Chun Liu, Guo N. Huang, Ge Tao

**Affiliations:** ^1^Shanghai Children's Medical Center, Shanghai Jiao Tong University School of Medicine, Shanghai, China; ^2^Department of Pathology and Laboratory Medicine, University of North Carolina at Chapel Hill, Chapel Hill, NC, United States; ^3^McAllister Heart Institute, University of North Carolina at Chapel Hill, Chapel Hill, NC, United States; ^4^Stanford Cardiovascular Institute, Stanford, CA, United States; ^5^Institute for Stem Cell Biology and Regenerative Medicine, Stanford, CA, United States; ^6^Department of Medicine (Division of Cardiology), Stanford University School of Medicine, Stanford, CA, United States; ^7^Cardiovascular Research Institute and Department of Physiology, University of California, San Francisco, San Francisco, CA, United States; ^8^Eli and Edythe Broad Center for Regeneration Medicine and Stem Cell Research, University of California, San Francisco, San Francisco, CA, United States; ^9^Department of Regenerative Medicine and Cell Biology, Medical University of South Carolina, Charleston, SC, United States

**Keywords:** regenerative medicine, cardiomyocytes, heart, developmental biology, cardiac maturation

Mature cardiomyocytes (CMs) are terminally differentiated cells that exit the cell cycle. The lack of adult CM progenitors makes it challenging to restore the loss of CMs caused by cardiac injury and disease. Current strategies to repopulate damaged cardiac tissue include exogenous cell-based or cell-free therapies (Chong et al., 2014) and reintroducing mature CMs into the cell cycle by genetic approaches (Ptaszek et al., 2012). However, adverse effects, such as arrhythmia and teratoma formation, were frequently observed in studies using pluripotent stem cell-derived CMs due to their immature nature. On the other hand, the discovery of the regenerative capacity in neonatal mouse CMs and the idea of rejuvenating mature CMs for therapeutic purposes sparked many studies to dissect the mechanism driving the maturation of CMs. This Research Topic has collected the most recent advances in the field of CM maturation.

The development of a mammalian heart is a multi-step process that includes cardiac lineage specification, morphogenesis, and maturation. CM maturation is initiated at mid-gestation and continues until adulthood. Compared to mechanisms of cardiac fate commitment in early embryonic development, the CM maturation process is not as well-defined (Moskowitz et al., 2007; Gupta and Poss, 2012; Del Monte-Nieto et al., 2018; Hu et al., 2018). To establish persistent and efficient contractility, CMs exit their cell cycles and undergo changes in cell structure, metabolism, and gene expression profile to reach maturation.

In mice, CM cell cycle exit occurs within the first postnatal week, while central cell-cycle-promoting networks are tightly repressed (Porrello et al., 2011; Mohamed et al., 2018). Cell cycle exit of mouse CMs is marked by polyploidization, resulting in karyokinesis without cytokinesis during the final round of cell proliferative activity. This leads to most mature mouse CMs containing two diploid nuclei (binucleation) (Li et al., 1996; Soonpaa et al., 1996). The postnatal CMs then enter a stage of hypertrophic growth in which the sarcomeres expand and reorganize to reach mature size. Furthermore, the reaction of developing CMs to pathological conditions attracted growing attention in the field. Ding et al. established a pulmonary artery banding (PAB) model in neonatal rats and mice. They showed that PAB accelerated the transition of mononuclear CMs into multinucleated cells to promote hypertrophic growth in neonatal hearts.

To propagate electrical activity into the CMs, transverse tubules (T-tubules) invaginate into the cells during postnatal development (Di Maio et al., 2007; Ziman et al., 2010). Unlike embryonic CM or induced CMs (iCMs) from iPSCs, mature ventricular CMs do not express HCN4 (hyperpolarization-activated cyclic nucleotide-gated potassium channel 4) and exhibit low automaticity (Kim et al., 2015). To establish simultaneous contraction, juvenile mouse CMs form junctions called intercalated discs (ICDs) between the ends of two CMs. Key markers of ICDs, including Connexin 43 and N-cadherin, are expressed in CMs from early development, specifically localizing to ICDs postnatally (Vreeker et al., 2014). However, the mechanisms of localizing ICD components to CM termini are not fully elucidated.

Another milestone of CM maturation is the shift from glycolysis to beta-oxidation to meet the high demand of ATP for cell contraction. Mitochondrial biogenesis increases until reaching a biomass that occupies 40% of the cell volume (Schaper et al., 1985). Unlike immature CMs, mature mitochondria contain densely organized cristae (Dai et al., 2017). This metabolic transition is regulated by multiple pathways such as Pparα and Nrf1/2 (Dorn et al., 2015; Uosaki et al., 2015).

The cellular microenvironment also plays a critical role in CM maturation. Manipulating *in vitro* cell culture environment and physical conditioning can promote CM maturation (Nunes et al., 2013; Zhang et al., 2013; Ronaldson-Bouchard et al., 2018). Evidence also revealed that subpopulations of non-CM cell types, including cardiac fibroblasts, endothelial cells, and macrophages, also drive CM maturation (Wang et al., 2020). Recently, Zhao H. et al. took a high-throughput approach to discover the critical transcription factors that induce cardiac reprogramming directly from injury-derived cardiac fibroblasts, providing a new recipe for the exciting field of cell-based therapy in heart regeneration. Morphological and structural development occurs along with the shift of key gene profiles such as isoform switches of sarcomere proteins, suggesting that the orchestration of multiple transcriptional regulatory pathways may control CM maturation. In this Research Topic, Fang et al. reported that T-Box Transcription Factor 20 (Tbx20) was activated in the myocardial wound edge in zebrafish to promote injury-induced CM proliferation. Tbx20 induced CM dedifferentiation, the loss of CM cellular contacts, and the re-expression of immature gene programs (Fang et al.). The authors also unexpectedly discovered that myocardial Tbx20 had a cell non-autonomous effect on endocardium expansion. In another study using zebrafish as a regenerative model, Peng et al. found that unlike the inhibitory function of the canonical Wnt pathway, wnt2bb-mediated non-canonical Wnt signals positively regulate CM proliferation.

Furthermore, Qi et al. studied the mechanism of inflammatory response in a diabetic cardiomyopathy model. They found that high glucose-induced lncRNA-MIAT is responsible for proinflammatory IL-17 production in CMs (Qi et al.). Cell-free therapy is another frontier in the field of heart regeneration and repair. Lyu et al. discovered that the 3'-untranslated region of Mcl1 transcript has a protective role in Ang II-induced cardiac apoptosis, shedding light on these novel therapeutic strategies.

Finally, Zhao M-T et al. provided an overview of the field, discussing how cardiac proliferation and maturation are regulated during embryonic development and postnatal growth, and exploring how patient iPSC-CMs could serve as the future seed cells for cardiac cell replacement therapy. Non-mammalian animals are particularly valuable inspirations for studies in regenerative medicine. Xia et al. reviewed the mechanisms of heart development and regeneration that were discovered in these models, highlighting the advantages of non-mammalian models as tools for cardiac research.

## Author Contributions

GT and MZ wrote the first draft. LQ, CL, and GH contributed key concepts to the editorial and revised the manuscript. All authors contributed to the article and approved the submitted version.

## Conflict of Interest

The authors declare that the research was conducted in the absence of any commercial or financial relationships that could be construed as a potential conflict of interest.

## Publisher's Note

All claims expressed in this article are solely those of the authors and do not necessarily represent those of their affiliated organizations, or those of the publisher, the editors and the reviewers. Any product that may be evaluated in this article, or claim that may be made by its manufacturer, is not guaranteed or endorsed by the publisher.
